# Impact of surgical strategies on the survival of gallbladder cancer patients: analysis of 715 cases

**DOI:** 10.1186/s12957-020-01915-7

**Published:** 2020-06-26

**Authors:** Yigang Chang, Qiang Li, Qian Wu, Limin Chi, Xiaogang Bi, Qingmin Zeng, Huaying Huo

**Affiliations:** 1 Department of Hepatobiliary Surgery, Tianjin Medical University Cancer Institute and Hospital, National Clinical Research Center for Cancer & Key Laboratory of Cancer Prevention and Therapy, Tianjin, Tianjin’s Clinical Research Center for Cancer, Tianjin, 300060 China; 2 Department of Gastrointestinal and Pancreas Surgery, Shanxi Provincial People’s Hospital, Taiyuan, 030012 China; 3 Department of Traditional Chinese Medicine, Shanxi Tumor Hospital, Taiyuan, 030013 China; 4 Physical Examination Center, First Hospital of Shanxi Medical University, Taiyuan, 030001 China; 5 Department of Gastrointestinal Surgery, Peking University Shougang Hospital, Beijing, 100041 China; 6 Department of Traditional Chinese Medicine, Shanxi Provincial People’s Hospital, Taiyuan, 030012 China

**Keywords:** Gallbladder cancer, Surgical strategy, Survival analysis, Radical surgery, Palliative surgery

## Abstract

**Objective:**

The aim of the study is to evaluate the impact of application of surgical strategies at different cancer stages on the survival of gallbladder cancer (GBC) patients.

**Methods:**

The patients with GBC were divided into 3 groups according to their received surgical strategies: simple resection (full-thickness cholecystectomy for removal of primary tumor site), radical resection (gallbladder bed removal combined with partial hepatectomy), and palliative surgery (treatment at advanced stages). The overall survival (OS) of GBC patients who were received different surgical strategies was compared.

**Results:**

Survival analysis showed that radical resection had a best OS at clinical stage II, and simple resection had a best OS at tumor clinical stage IV. Cox hazard proportional regression analysis showed that more advanced tumor stages, tumor location of gallbladder body or neck, and CA199 ≥ 27 U/mL were the major risk factors for the OS of GBC.

**Conclusions:**

At tumor stage II, radical resection should be the most effective surgical therapy for GBC. However, the effect of radical resection at advanced stages could be restricted. The utilization of radical resection should be increased at tumor stage II for a better long-term survival outcome.

## Introduction

According to the Global Cancer Statistics 2018, gallbladder cancer (GBC) caused an estimated 219,420 new cases and 165,087 new deaths worldwide in the year 2018 [1]. The incidence and mortality of GBC is much higher in developing countries or areas, such as India, Valdivia, Chile, South America, Pakistan, Eastern Europe, and China, than that in developed countries [2]. As the most common cancer type of biliary tract cancer, 70% of GBC are incidentally diagnosed after a routine cholecystectomy for a benign disease [3]. Due to the lack of specific protocol for clinic diagnosis in early stages, GBC is usually found at advanced stages [4]. The outcome of GBC is poor, with a less than 5% of overall 5-year survival; however, 75% of overall 5-year survival can be achieved if the cancer is detected at an early stage [5]. Multiple factors, including environment, diagnosis, tumor stages, treatment options, and other complications, can impact the survival outcome of GBC patients [6–8].

Guidelines for GBC radical resection vary in different countries [9]. Goetze et al. have demonstrated the effective of radical resection for incidental GBC [5]. However, as an important treatment strategy of GBC, radical resection is underutilized in practice [10, 11]. According to the Chinese Guidelines for GBC, the surgical treatment is suggested based on the TNM staging: simple cholecystectomy is recommended in patients with a tumor at Tis or T1a stage; radical resection is recommended in patients with stage T1b or T2; palliative treatment is suggested in patients at T3 or T4 stage. The suggested treatment of radical resection for GBC is wedge resection of the gallbladder bed with no less than 2 cm margin in the liver, a resection of liver segments 4b and 5, or a resection of the right liver, which is combined with dissection of the regional lymph nodes. For advanced stages of GBC, palliative treatment, such as palliative cholecystectomy, percutaneous transhepatic cholangial drainage (PTCD), percutaneous transhepatic biliary drainage (PTBD), and gallbladder bypass, is also recommended. Since liver resection has been verified to have a poor survival outcome, with a 13~18% in-hospital mortality, [12, 13], the effect of radical surgery for GBC patients at advanced stages with poor conditions is quite limited. Kawahara et al. also have reported an effective surgical strategy for extent of resection in GBC at T2 stage, which is based on the gallbladder location of tumor [14].

Full-thickness cholecystectomy, radical resection surgery, and palliative surgery are the major surgical strategies for GBC at present. Few studies have systematically compared the application of different surgical strategies for GBC. Here, we aimed to analyze the OS of 715 GBC patients who have received full-thickness cholecystectomy, radical resection surgery, or palliative surgery and investigate the impact of strategy of surgery on the long-term survival in GBC patients at different tumor stages.

## Methods

### Patients

All patients with GBC in the present study received anti-tumor treatment in the Tianjin Medical University Cancer Institute and Hospital, the Shanxi Provincial People’s Hospital, the Shanxi Tumor Hospital, or the First Hospital of Shanxi Medical University from 2012 to 2017. The initial symptoms of the cancer patients mainly include abdominal discomfort (~ 50%) and jaundice (~ 28%), and about 14% patients were detected by physical examination as well. Then, the patients were further diagnosed by B-ultrasound (BUS), computed tomography (CT), pathology, and magnetic resonance imaging (MRI). All patients were staged according to the 8th edition of the AJCC/UICC classification in 2017 [15]. Patients accompanied with other malignant tumor, patients without determined clinical pathology, patients with recurring GBC, and patients without surgical treatment were excluded. Patients were divided into three groups according to the strategies of surgical treatment received: simple resection, radical resection, and palliative surgery. Simple resection was defined as “partial or total resection of primary tumor site,” and radical resection was defined as “total resection of primary tumor site with other organs”; palliative surgery was performed in patients with distant metastases cancer, wide tumor invasion, and conditions wherein they cannot bear aggressive surgery or they refuse.

### Demographics and clinicopathological information of patients

A total of 715 patients were recruited in the study. Demographics (source, age, gender, and BMI) and clinicopathological (tumor grade, clinical staging, TNM stage, gallbladder location of tumor, smoking or not, CA199, CA242, with chronic disease or gallstone or not, adjuvant therapy, strategy of surgery) information of the GBC patients were collected for the analysis. Gallbladder location of tumor was grouped as follows: fundus of the gallbladder, body of the gallbladder, and neck of the gallbladder or unknown. Involved chronic diseases mainly included diabetes, hypertension, and coronary heart disease. The adjuvant therapy mainly included radiotherapy, chemotherapy, and biotherapy.

### Statistical analysis

All data in present study were analyzed using MedCalc Statistical Software version 15.2.2 (MedCalc Software bvba, Ostend, Belgium). Discrete variables were presented as number and percentage. Statistical difference analysis in baseline characteristics was performed using chi-squared (*χ*^2^) test. Overall survival (OS) curves for GBC patients were assessed using Kaplan-Meier survival method, and the Logrank test was used to compare the difference between subgroups. Univariate and multivariate Cox proportional hazards regression analysis was performed to reveal the risks factors for GBC survival outcomes. *p* < 0.05 indicates statistically significant difference.

## Results

### Population and characteristics of the GBC patients

There were a total of 1146 cases of diagnosed GBC from 2012 to 2017. Three hundred ninety-three cases were excluded for undergoing no surgery or an unknown surgery, 18 cases were excluded for recurrence of primary GBC, and 20 cases were excluded for suffering with other types of cancer. Of the enrolled 715 cases, 126 (17.5%) cases received simple resection, 349 (48.8%) cases underwent radical resection, and 240 (35.6%) cases were treated with palliative surgery. Patients’ general and clinicopathologic information were shown in Table 1. The median age of the patients was 62 years (min/max age = 29/88 years). Two hundred fifty-eight (36.1%) females and 457 (63.9%) males were included in the recruited population. Two hundred ninety-nine (41.8%) patients were from the urban, and 416 (58.2%) patients were from the country.

**Table 1:** General, clinicopathologic, treatment, and follow-up characteristics for the 715 gallbladder cancer patients with different surgery treatments

Variates	Total	Simple resection	Radical resection	Palliative surgery	*p*
	(*n* = 715) *N* (%)	(*n* = 126) *N* (%)	(*n* = 349) *N* (%)	(*n* = 240) *N* (%)	
Sources					0.2488
Urban	299 (41.8)	56 (44.4)	153 (43.8)	90 (37.5)	
Country	416 (58.2)	70 (55.6)	196 (56.2)	150 (62.5)	
Ages (years)					0.0198
< 60	275 (38.5)	41 (32.5)	154 (44.1)	80 (33.3)	
60–69	264 (36.9)	48 (38.1)	125 (35.8)	91 (37.9)	
≥ 70	176 (24.6)	37 (29.4)	70 (20.1)	69 (28.8)	
Sex					0.0573
Female	258 (36.1)	34 (27)	130 (37.2)	94 (39.2)	
Male	457 (63.9)	92 (73)	219 (62.8)	146 (60.8)	
BMI (Kg/m^2^)					0.293
< 28	613 (85.7)	109 (86.5)	301 (86.2)	203 (84.6)	
≥ 28	80 (11.2)	10 (7.9)	39 (11.2)	31 (12.9)	
Unknown	22 (3.1)	7 (5.6)	9 (2.6)	6 (2.5)	
Smoking history					0.3083
No	568 (79.4)	105 (83.3)	279 (80)	184 (76.7)	
Yes	147 (20.6)	21 (16.7)	70 (20)	56 (23.3)	
Gallbladder location of tumor					< 0.0001
Fundus	208 (29.1)	45 (35.7)	123 (35.2)	40 (16.7)	
Body	321 (44.9)	62 (49.2)	151 (43.3)	108 (45)	
Neck	150 (21)	19 (15.1)	75 (21.5)	56 (23.3)	
Unknown	36 (5)	0	0	36 (15)	
Clinical staging					< 0.0001
I	35 (4.9)	20 (15.9)	15 (4.3)	0	
II	82 (11.5)	37 (29.4)	45 (12.9)	0	
III	291 (40.7)	59 (46.8)	214 (61.3)	18 (7.5)	
IV	307 (42.9)	10 (7.9)	75 (21.5)	222 (92.5)	
T stage					< 0.0001
T1	34 (4.8)	16 (12.7)	18 (5.2)	0	
T2	92 (12.9)	38 (30.2)	53 (15.2)	1 (0.4)	
T3	381 (53.3)	64 (50.8)	255 (73.1)	62 (25.8)	
T4	125 (17.5)	4 (3.2)	22 (6.3)	99 (41.3)	
Unknown	83 (11.6)	4 (3.2)	1 (0.3)	78 (32.5)	
N stage					< 0.0001
N0	276 (38.6)	70 (55.6)	197 (56.4)	9 (3.8)	
N1	88 (12.3)	5 (4)	77 (22.1)	6 (2.5)	
N2	90 (12.6)	3 (2.4)	58 (16.6)	29 (12.1)	
Unknown	211 (29.5)	48 (38.1)	17 (4.9)	146 (60.8)	
M stage					< 0.0001
M0	524 (73.3)	119 (94.4)	338 (96.8)	67 (27.9)	
M1	158 (22.1)	6 (4.8)	8 (2.3)	144 (60)	
Unknown	33 (4.6)	1 (0.8)	3 (0.9)	29 (12.1)	
Grade					< 0.0001
Well-differentiated	27 (3.8)	13 (10.3)	11 (3.2)	3 (1.3)	
Moderately differentiated	112 (15.7)	30 (23.8)	73 (20.9)	9 (3.8)	
Poorly differentiated	184 (25.7)	28 (22.2)	122 (35)	34 (14.2)	
Unknown	392 (54.8)	55 (43.7)	143 (41)	194 (80.8)	
CA199					0.0266
< 27 U/mL	262 (36.6)	59 (46.8)	168 (53.2)	35 (14.6)	
≥ 27 U/mL	388 (54.3)	49 (38.9)	158 (45.3)	181 (75.4)	
Unknown	65 (9.1)	18 (14.3)	23 (6.6)	24 (10)	
CA242					
< 20 IU/mL	296 (41.4)	61 (48.4)	171 (49)	64 (26.7)	< 0.0001
≥ 20 IU/mL	311 (43.5)	33 (26.2)	131 (37.5)	147 (61.3)	
Unknown	108 (15.1)	32 (25.4)	47 (23.5)	29 (12.1)	
With chronic disease					0.2935
No	433 (60.6)	74 (58.7)	204 (58.5)	155 (64.6)	
Yes	282 (39.4)	52 (41.3)	145 (41.5)	85 (35.4)	
Gallstone					< 0.0001
No	321 (44.9)	34 (27)	162 (46.4)	125 (52.1)	
Yes	394 (55.1)	92 (73.1)	187 (53.6)	115 (47.9)	
Adjuvant therapy					< 0.0001
None	537 (75.1)	96 (76.2)	239 (68.5)	202 (84.2)	
Received	178 (24.9)	30 (23.8)	110 (31.5)	38 (15.8)	
Follow-up					
Alive	212 (29.7)	59 (46.8)	136 (39)	17 (7.1)	
3-year survival	254 (35.5)	69 (54.8)	134 (38.4)	51 (21.3)	
Months, median(95% CI)	27 (22–31)	51 (41–69)	34 (28–44)	10 (7–14)	

Results are number (%) unless otherwise specified

### Association between the characteristic factors and surgical strategies

Chi-squared (*χ*^2^) analysis showed that there was a significant difference among the three groups including ages (*p* < 0.05), gallbladder location of tumor (*p* < 0.0001), clinical staging (*p* < 0.0001), TNM stage (*p* < 0.0001), tumor grade (*p* < 0.0001), CA199 (*p* < 0.05), CA242 (*p* < 0.0001), gallstone (*p* < 0.0001), and adjuvant therapy (*p* < 0.0001). In detail, patients with tumor location not in gallbladder neck, earlier clinical staging (I/II), T1/T2 stage, normal level of tumor markers, and gallstone were more likely to undergo simple resection. Patients with young age, N1/N2 stage, and poorly differentiated tumor were more likely to receive radical resection. Patients with M1 stage, CA199 ≥ 27 U/ml, CA242 ≥ 20 IU/ml, and unreceived adjuvant therapy were more likely to receive palliative surgery.

### The impact of surgical strategies on OS of patients

**Fig. 1:**
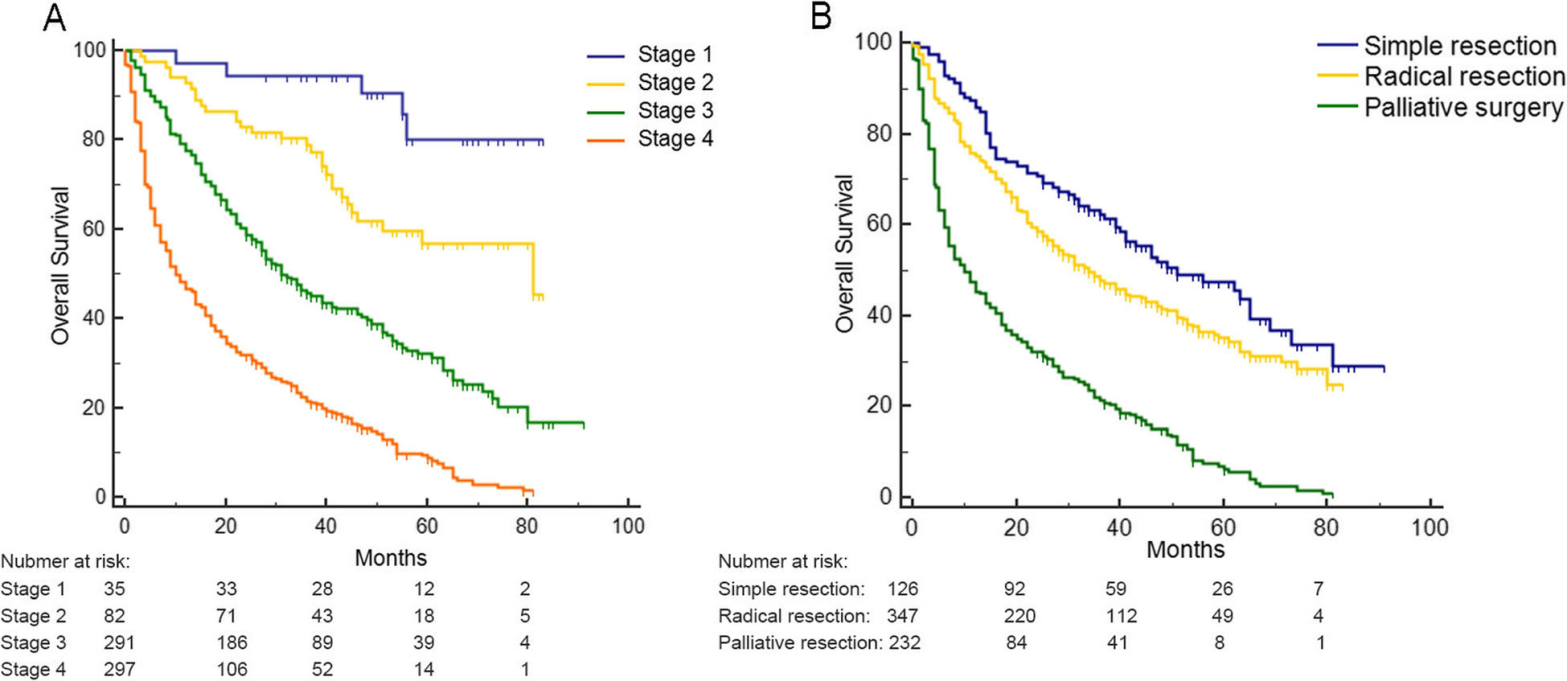
Overall survival (OS) for GBC according to stages and surgical strategies. **a** Kaplan-Meier survival analysis was used to compare OS among patients with different cancer stages (*p* < 0.0001). **b** Kaplan-Meier survival analysis was used to compare OS of patients received with different surgical strategies (*p* < 0.0001)

The median OS time of the 715 patients was 24 months (95% CI 22–28 months). Kaplan-Meier survival analysis was performed to measure OS with different clinical stages and different surgical strategies, respectively (Fig. 1). Form stage I to IV cases, the survival rate were 85.71%, 64.63%, 36.08%, and 10.42% (Fig. 1a). As shown in Fig. 1b, compared with the palliative surgery groups, patients with simple resection or radical resection had significant longer OS time (*p* < 0.0001). Patients with simple resection had the best OS outcome, with a 47.62% of survival rate and 51 months of median OS time (95% CI 41–69 months). The OS outcome of patients with radical resection was moderate, with a 39.83% of survival rate and 34 months of median overall survival time (95% CI 28–44 months). The palliative surgery patient group had the worst OS outcome, with an 8.75% of survival rate and 10 months of median OS time (95% CI 7–14 months).

**Fig. 2:**
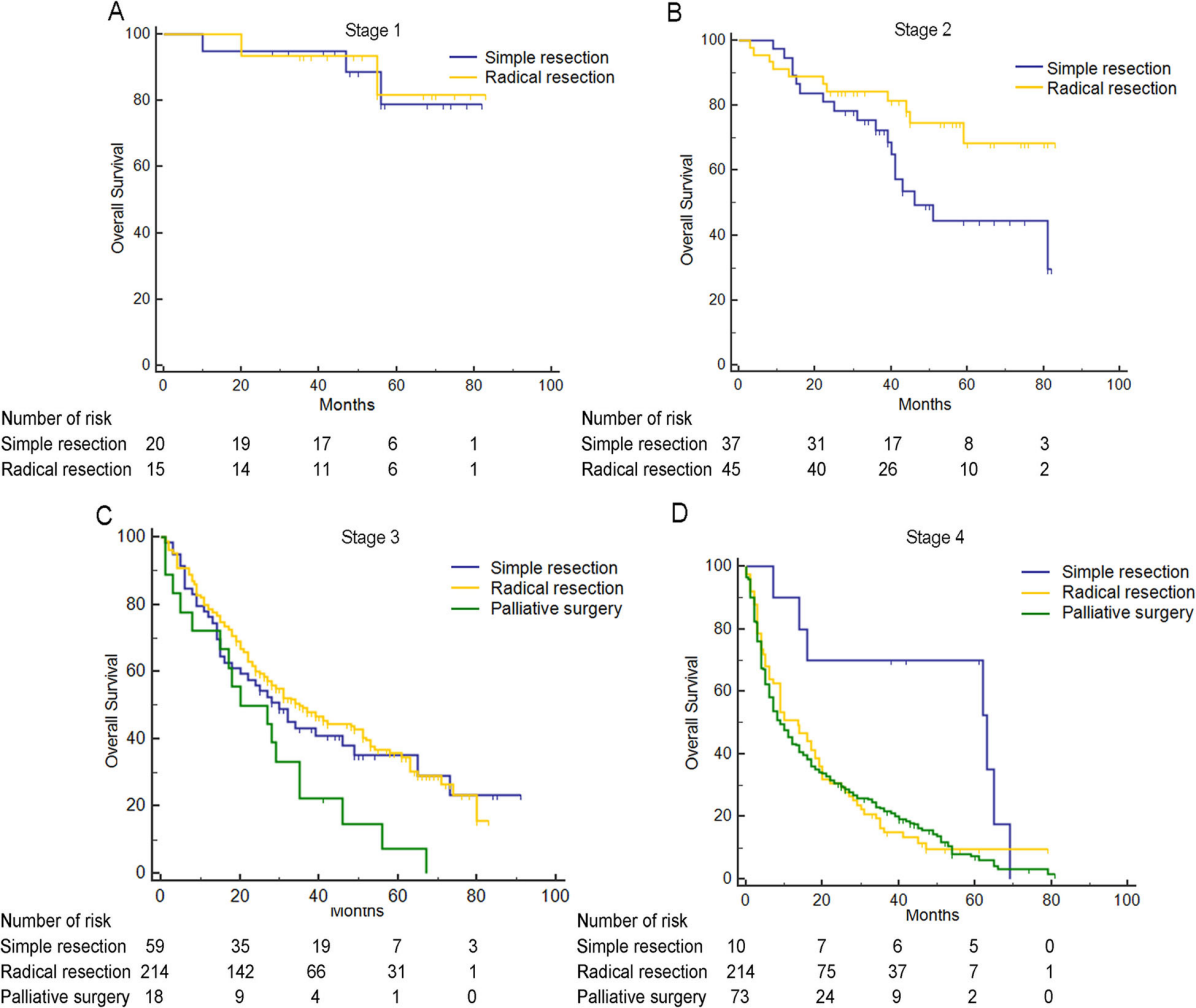
OS for GBC at different stages according to surgical strategies. **a** OS for stage I (*p* = 0.9344). **b** OS for stage II (*p* = 0.0415). **c** OS for stage III (*p* = 0.0275). **d** OS for stage IV (*p* = 0.0129)

Furthermore, the effect of the surgical strategies on different cancer clinical stages were explored. The difference of OS between simple resection and radical resection in stage I patients was not significant (*p* = 0.9344, Fig. 2a). However, patients with radical resection had a better OS than patients with simple resection at stage II (*p* = 0.0415, Fig. 2b). Compared with simple resection and radical resection groups, patients with palliative surgery had a worse overall survival at cancer stage III (*p* = 0.0275, Fig. 2c). At cancer stage IV, patients with simple resection also had a best overall survival (*p* = 0.0129, Fig. 2d).

### The risk factors associated with the OS

Univariate and multivariate Cox proportional hazards regression model was established to evaluate the hazard ratio (HR) of variates (Table 2). Univariate analysis showed that patients from the countryside, with tumor location of gallbladder body or neck, with increased tumor stages, with increased TNM stages, with poorly differentiated of the tumor, with CA199 ≥ 27 U/mL, with CA242 ≥ 20 IU/mL, and with surgical treatment of radical resection or palliative surgery were related to a worse long-term survival of GBC. Multivariate analysis showed that the tumor location of gallbladder body or neck (HR = 1.4901, 95% CI = 1.1858 to 1.8726, *p* = 0.007), more advanced tumor stages, and CA199 ≥ 27 U/mL (HR = 1.3941, 95% CI = 1.0838 to 1.7933, *p* = 0.0101) might be the main causes for induction a worse long-term survival of GBC, and the surgical strategies of radical resection (HR = 1.0397, 95% CI = 0.7820 to 1.3823, *p* = 0.7898) or palliative surgery (HR = 1.41, 95% CI = 0.9886 to 2.0111, *p* = 0.0592) were not associated with OS.

**Table 2:** Cox proportional hazards regression analysis for OS

Variates	Univariate analysis			Multivariate analysis		
	HR	95% CI	*p*	HR	95% CI	*p*
Sources						
Urban	Ref	Ref	Ref	Ref	Ref	Ref
Country	1.221	1.0212–1.4598	0.0293	1.0272	0.8533–1.2364	0.7779
Ages (years)						NI
< 60	Ref	Ref	Ref			
60–69	0.9238	0.7541–1.1316	0.4461			
≥ 70	1.0349	0.8269–1.2952	0.08909			
Sex						NI
Female	Ref	Ref	Ref			
Male	0.883	0.7374–1.0573	0.1781			
BMI (Kg/m^2^)						NI
< 28	Ref	Ref	Ref			
≥ 28	1.1157	0.8557–1.4548	0.4211			
Unknown	0.4844	0.2595–0.9043	0.0236			
Smoking history						NI
No	Ref	Ref	Ref			
Yes	1.0663	0.8604–1.3215	0.5596			
Gallbladder location of tumor						
Fundus	Ref	Ref	Ref	Ref	Ref	Ref
Body	1.5472	1.2442–1.9241	0.0001	1.4901	1.1858–1.8726	0.0007
Neck	1.4257	1.0976–1.8519	0.0082	1.1776	0.8959–1.5479	0.2437
Unknown	1.445	0.9751–2.1412	0.068	0.6761	0.4434–1.0310	0.0705
Clinical staging						
I	Ref	Ref	Ref	Ref	Ref	Ref
II	2.9912	1.1632–7.6921	0.0237	2.9253	1.1281–7.5861	0.0281
III	7.3437	3.0318–17.7882	< 0.0001	6.4359	2.6198–15.8108	0.0001
IV	16.4044	6.7898–39.6340	< 0.0001	10.4364	4.1505–26.2422	< 0.0001
T stage						NI
T1	Ref	Ref	Ref			
T2	2.2162	0.9901–4.9607	0.0541			
T3	5.6811	2.6900–11.9979	< 0.0001			
T4	9.2513	4.3199–19.8121	< 0.0001			
Unknown	12.0606	5.5666–26.1303	< 0.0001			
N stage						NI
N0	Ref	Ref	Ref			
N1	2.4167	1.7968–3.2505	0.017			
N2	4.2985	3.2423–5.6989	< 0.0001			
Unknown	3.1367	2.5164–3.9099	< 0.0001			
M stage						NI
M0	Ref	Ref	Ref			
M1	2.4876	2.0433–3.0286	< 0.0001			
Unknown	1.4786	1.0065–2.1721	0.0474			
Grade						
Well-differentiated	Ref	Ref	Ref	Ref	Ref	Ref
Moderately differentiated	1.4133	0.7651–2.6107	0.2717	1.3022	0.6944–2.4420	0.4128
Poorly differentiated	2.3874	1.3269–4.2957	0.0039	1.7171	0.9330–3.1604	0.0839
Unknown	2.4023	1.3517–4.2694	0.003	1.5552	0.8560–2.8257	0.1493
CA199						
< 27 U/mL	Ref	Ref	Ref	Ref	Ref	Ref
≥ 27 U/mL	2.2785	1.8655–2.7830	< 0.0001	1.3941	1.0838–1.7933	0.0101
Unknown	1.3304	0.9374–1.8881	0.1118	1.2309	0.6981–2.1705	0.475
CA242						
< 20 IU/mL	Ref	Ref	Ref	Ref	Ref	Ref
≥ 20 IU/mL	1.9779	1.6322–2.3969	< 0.0001	1.1169	0.8790–1.4192	0.3679
Unknown	1.042	0.7776–1.3964	0.7838	1.0146	0.6312–1.6310	0.9525
With chronic disease						NI
No	Ref	Ref	Ref			
Yes	0.8733	0.7292–1.0460	0.1432			
Gallstone						NI
No	Ref	Ref	Ref			
Yes	0.9403	0.7885–1.1213	0.4955			
Adjuvant therapy						NI
None	Ref	Ref	Ref			
Received	0.8719	0.7105–1.0699	0.1916			
Surgery						
Simple resection	Ref	Ref	Ref	Ref	Ref	Ref
Radical resection	1.3679	1.0384–1.8021	0.0267	1.0397	0.7820–1.3823	0.7898
Palliative surgery	3.3214	2.5191–4.3792	< 0.0001	1.41	0.9886–2.0111	0.0592

*Ref* reference, *CI* confidence interval, *NI* not included in the multivariate survival analysis

## Discussion

GBC is an uncommon cancer type with a high mortality rate and poor long-term survival outcomes [16]. Surgical treatment is the most effective intervention for the cure of GBC patients [17]; however, curative resection is feasible in minority population of GBC patients [18]. According to the Guidelines of the National Comprehensive Cancer Network, a radical resection is recommended for T1b and more advanced GBC [19]. Actually, the option for surgical strategies should be confirmed according to both the patients’ conditions and tumor clinicopathology characteristics [20]. On the other hand, Goetze et al. have reported the phenomenon of that compared with following the guidelines; the application of radical resection for incidental GBC much more depend on the volume of clinical liver surgery in Germany [9]. Thus, we suspected that the options of surgery strategies in GBC patients may affect the overall survival outcomes of cancer.

In the present study, the surgical strategies for GBC were divided into 3 subgroups, including simple resection, radical resection, and palliative surgery. Among these investigated categories, we found that ages, tumor clinical stages, TNM stages, tumor grade, gallbladder location of tumor, tumor markers (CA199 and CA242), gallstone, and adjuvant therapy were the influencing factors for surgery strategies. Patients with earlier tumor clinical stage, earlier T stage, tumor location not in gallbladder neck, normal level of tumor markers, and gallstone were more associated with simple resection. Analogously, a study in 87 GBC patients by Wang et al. has reported that the levels of tumor markers (CA199, CA125, and CA242) in patients with tumor in gallbladder neck were much higher than in those with tumor in gallbladder fundus and body [21]. Patients with young ages, N1/N2 stage, and poorly differentiated were more associated with radical resection. It has been proved that liver resection is related to a high mortality rate, and young age would play a protective role for patients with radical resection in perioperative period. Patients with advanced tumor stages, high levels of tumor markers (CA199, CA242), metastatic cancer (M1), and adjuvant therapy unreceived were more associated with palliative surgery. For the metastatic cancer and surgery inoperable patient, palliative surgery would be the treatment for relieving the patient’s pain and promoting patients’ quality of life.

As our before results, surgical strategies were closely associated with the tumor stages, and the long-term survival outcome of GBC patients is distinguished according to the stage-adjusted surgical strategies (Fig. 1b). Plenty of studies have indicated that the performance of radical resection might be the best choice for GBC management at clinical stage II [14, 17, 22–25]. In our study, we also found that radical resection should be the only reliable and safe surgical therapy for GBC patients with stage II (Fig. 2b). However, a high mortality rate within about 12 months after surgery might be one of the primary limitations for the utilization of radical surgery at stage II. At advanced stages, compared with simple resection or palliative surgery, the effect of radical surgery on overall survival was obviously decreased (Fig. 2c, d). Previous studies have assessed the efficiency of radical surgery at stages III and IV, with a 25 to 50% 5-year survival [17, 26, 27]. However, the comparison among different surgical strategies at advanced stages is rare. In current study, there is no significant difference in 5-year survival at stage III between radical resection and simple resection groups, but palliative surgery groups had a lowest overall survival. Thus, aggressive resection is still an effective therapy at stage III, even if it is available in partial individuals. The application of radical resection in stage IV patients is controversial. In our study, we also found that the difference of overall survival at stage IV between radical resection and palliative surgery groups was not significant (Fig. 2d). Also, it seemed that the simple resection groups had a good performance for overall survival at stage IV; however, the subgroup size was too small to summarize a reliable conclusion.

At present, the prognosis of GBC mainly depends on the tumor stages [22]. The Cox hazard proportional regression analysis showed that advanced tumor stages, high levels of CA199, and tumor location in gallbladder body or neck would indicate a poor prognosis. Studies have reported that CA199 is a prognosis related marker [21, 28, 29]. Moreover, Wang et al. have reported that tumor location in gallbladder neck could be a prognosis-related marker [21]. Compared with aggressive resection, palliative surgery groups would have a worse prognosis (HR = 1.41, 95% CI = 0.9886 to 2.0111, *p* = 0.0592).

However, there were some limitations in our study. In the investigated population, more than 70% of them were patients with advanced cancer, in which the effect of surgical treatment is quite limited. Also, since the outcome for advanced GBC is poor, the willingness of patients with cancer at stage IV for aggressive therapy is usually wobbly, which may impact the strategic choice for surgery to a large extent. On the other hand, the surveyed sample size was limited; the long-term survival come for GBC could be affected by composite factors [6, 30, 31].

In conclusion, the overall survival for GBC mainly depends on the stages of detected tumor; however, aggressive surgery could be always the reasonable surgical therapy for patients with GBC, especially, and radical resection could be a most effective surgical strategy for patients with tumor at stage II to obtain a long-term survival. Additionally, our report supports the viewpoint that the role of radical resection in advanced stages is restricted, but, in early stage, the utilization of radical surgery should be further developed.

## Data Availability

The datasets used or analyzed during the current study are available from the corresponding author on reasonable request.
